# *Copy of a Letter from the* Earl of Westmeath *to* Dr. Jenner, *Dated May* 23, 1805

**Published:** 1805-09-01

**Authors:** 


*Copy of a Letter from the* EARL OF WESTMEATH *to* DR. JENNER, *dated May* 23, 1805.

SIR,

UNDERSTANDING that a report has been circulated, which, if believed, would tend much to weaken that confidence, which is at present so generally and so justly entertained by the public, in your system of inoculation for the cow-pox, namely, that my youngest son had taken the natural small-pox, after having been vaccinated, I think it but justice to you to contradict the report; and to state, for your satisfaction, the real circumstances of the case, which are as follow:

When he was about two months old, he was inoculated for the small-pox, in the Suttonian method, by a physician in Ireland, who has been very generally successful in inoculation, and pronounced by him to be perfectly free from the risk of infection; notwithstanding which, he caught the infection, about a fortnight since, and is now recovering from the natural small-pox.

I beg to inform you, at the same time, that my youngest daughter, who was vaccinated by you, about four years since, has not only been frequently exposed to the danger of infection, but was actually inoculated for the *small-pox*, without taking it. I have considered it incumbent on me to bear testimony to the efficacy of the vaccine system, as I consider the report relative to my son, which originated in misrepresentation, to have been circulated for purposes obviously prejudicial to that most useful and fortunate discovery.

I request you will make any use of this communication, which you may think necessary.

I am, Sir,
Your obedient humble servant,
WESTMEATH.

